# Future hydrological constraints of the Montseny brook newt (*Calotriton arnoldi*) under changing climate and vegetation cover

**DOI:** 10.1002/ece3.5506

**Published:** 2019-08-01

**Authors:** José L. J. Ledesma, Albert Montori, Vicent Altava‐Ortiz, Antonio Barrera‐Escoda, Jordi Cunillera, Anna Àvila

**Affiliations:** ^1^ Center for Advanced Studies of Blanes Spanish National Research Council Blanes Spain; ^2^ Department of Aquatic Sciences and Assessment Swedish University of Agricultural Sciences Uppsala Sweden; ^3^ GRENP (Grup de Recerca de l'Escola de la Natura de Parets del Vallès), Life‐Tritó del Montseny Diputació de Barcelona Parets del Vallès Spain; ^4^ Department of Applied Research and Modelling Meteorological Service of Catalonia Barcelona Spain; ^5^ Department of Climatology Meteorological Service of Catalonia Barcelona Spain; ^6^ CREAF Campus de Bellaterra (UAB) Cerdanyola del Vallès Spain

**Keywords:** amphibians, Calotriton arnoldi conservation, catchment management, endangered species, environmental change, Mediterranean climate, PERSiST model, statistical downscaling

## Abstract

The Montseny brook newt (*Calotriton arnoldi*) is a critically endangered amphibian species which inhabits a small 20 km^2^ holm oak and beech forest area in NE Spain. *Calotriton arnoldi* strictly lives in running waters and might be highly vulnerable to hydrological perturbations expected to occur under climate and vegetation cover changes. Knowledge about the potential response of the species habitat to environmental changes can help assessing the actions needed for its conservation. Based on knowledge of the species and supported by observations, we proposed daily low and high streamflow event thresholds for the viability of *C. arnoldi*. We used the rainfall–runoff model PERSiST to simulate changes in the frequency and duration of these events, which were predicted under two climate and four vegetation cover scenarios for near‐future (2031–2050) and far‐future (2081–2100) periods in a reference catchment. All future scenarios projected a significant decrease in annual streamflow (from 21% to as much as 67%) with respect to the reference period. The frequency and length of low streamflow events will dramatically increase. In contrast, the risk of catastrophic drift linked to high streamflow events was predicted to decrease. The potential change in vegetation toward an expansion of holm oak forests will be more important than climate changes in determining threshold low flow conditions. We thus demonstrated that consideration of potential changes in vegetation and not only changes in climate variables is essential in simulating future streamflows. This study shows that future low streamflow conditions will pose a severe threat for the survival of *C. arnoldi* and may help taking management actions, including limiting the expansion of holm oak forest, for ameliorating the species habitat and help its conservation.

## 1 INTRODUCTION

**Figure 1:**
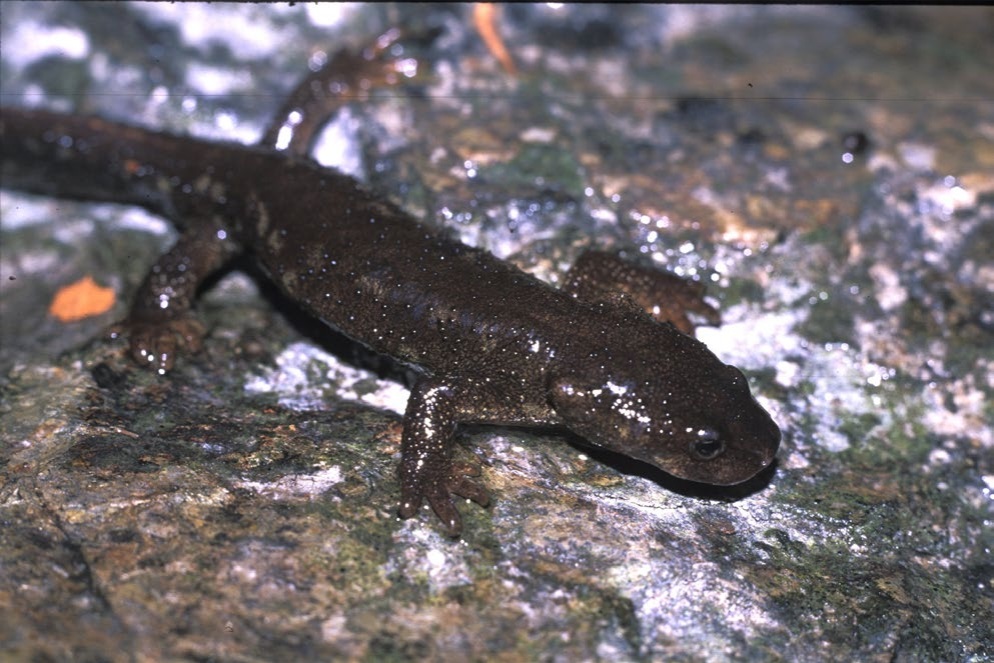
Adult individual of Montseny brook newt (*Calotriton arnoldi*) in its natural habitat

The Montseny brook newt (*Calotriton arnoldi*; Figure 1) was first described in 2005 as a new amphibian taxon in the Montseny mountains, 40 km NE of Barcelona (Spain) (Carranza & Amat, 2005). Phylogenetic analysis based on 12S and 16S DNA sequences indicated that the newt populations from Montseny had evolved separately from the Pyrenean brook newt (*Calotriton asper*) populations for more than one million years and now they constitute two clearly differentiated species (Valbuena‐Ureña, Amat, & Carranza, 2013). *Calotriton arnoldi* is found in only seven locations distributed in a small 20‐km^2^ area, and catchment divides appear to constitute a natural barrier that hampers interbreeding between populations (Carranza & Amat, 2005). Because of this lack of connectivity and the low population densities (an estimated total of ca. 2,000 adults), *C. arnoldi* is highly endangered and it is included in the IUCN Red List of Threatened Species. The *Diputació de Barcelona* was awarded a LIFE project (LIFE‐Tritó Montseny) in 2016 devised to protect *C. arnoldi*. Among others, project objectives included increasing the scientific knowledge of the ecological requirements of the species and assessing possible threats that may affect it. There is still little knowledge about the species ecological requirements and its habitat potential response to environmental changes. This information is of paramount importance to guide efforts for its conservation.

*Calotriton arnoldi* is found in oligotrophic, cold, fast running waters draining typical holm oak (*Quercus ilex*) and beech (*Fagus sylvatica*) forests in Montseny and it appears to completely depend on aquatic resources, as neither larvae nor adults have been found to inhabit the terrestrial environment. Its remarkable morphological traits, including reduced lung volume that may reduce buoyancy, and its thin skin provide further evidence of its adaptation to the aquatic environment (Valbuena‐Ureña et al., 2013). Therefore, drying out of headwater streams will pose a serious risk for the species, either directly because of the unfavorable habitat conditions, or indirectly due to the need of the individuals to move to wetted refuge areas in streambanks or to the hyporheic zone. Prey abundance in these refuge microsites may be very low, potentially impairing an appropriate development of the individuals (Clergue‐Gazeau, 1969, 1971). Other hydrological threats concern just the opposite, the risks associated with very high flows. Newts and other organisms inhabiting running waters are highly vulnerable to high streamwater velocity and flooding (Walls, Barichivich, & Brown, 2013). These events have been reported to cause catastrophic drift in *C. asper* (Colomer, Montori, García, & Fondevilla, 2014; Montori et al., 2012).

Consequently, *C. arnoldi* might be highly vulnerable to hydrological perturbations, either linked to low flows during dry periods or linked to high hydraulic discharge associated with extreme precipitation events. Both situations may be more frequent in a world undergoing climate change. Precipitation has not shown a clear trend in recent decades in NE Iberian Peninsula (González‐Hidalgo, López‐Bustins, Štepánek, Martín‐Vide, & Luisa, 2009; del Río, Herrero, Fraile, & Penas, 2011), but air temperature is rising (Vicente‐Serrano et al., 2014) and, particularly, the Montseny massif has experienced an increase of around 0.3°C per decade in the second half of the 20th century (Peñuelas & Boada, 2003). A temperature increase can lead to enhanced evapotranspiration (Wang, Dickinson, & Liang, 2012), which eventually will result in reduced streamflow. Besides climate, landscape cover type has also an influence on water resources. Several catchment studies have shown that an expansion of forest cover leads to a decrease in water yield (Bosch & Hewlett, 1982; Gallart & Llorens, 2004). In Montseny, the holm oak forest appears to be expanding with altitude at the expense of beech forests and heathlands (Peñuelas & Boada, 2003), which may further contribute to reduce streamflow by increasing evapotranspiration. Thus, both climate and vegetation cover changes may threaten *C. arnoldi* populations. However, so far no studies have quantified the magnitude, duration, and frequency of future hydrological extremes in the region that can affect the species survival. Hydrological consequences of climate and vegetation changes are better explored at the catchment scale using rainfall–runoff models, which can be calibrated against observed streamflow (Lupon, Ledesma, & Bernal, 2018; Oni et al., 2016; Seibert & McDonnell, 2010).

Here, we used the rainfall–runoff model PERSiST (Precipitation, Evapotranspiration and Runoff Simulator for Solute Transport; Futter et al., 2014) to investigate how changes in herein proposed daily low and high hydrological thresholds might threat populations of *C. arnoldi*. Streamflow was simulated at two future intervals (near‐future 2031–2050 and far‐future 2081–2100) under two climate scenarios (RCP4.5 and RCP8.5) in a reference catchment in Montseny. The hydrological effects of four plausible vegetation cover scenarios were also considered: (i) vegetation as present, (ii) displacement of holm oak and beech into heathland, (iii) expansion of holm oak over the beech but similar heathland cover, and (iv) holm oak covering the whole catchment. The relative role of climate versus vegetation cover in future hydrological extremes threatening *C. arnoldi* was also examined. Finally, management implications were discussed.

## 2 MATERIALS AND METHODS

### 2.1 Catchment selection and characterization

Because *C. arnoldi* is catalogued as critically endangered in the IUCN Red List of Threatened Species, providing details on specific locations where populations can be found is not advised in the context of the Life‐Tritó Montseny project. The *Diputació de Barcelona* is developing strict plans to ensure the survival of *C. arnoldi*, and this information is considered sensitive. For this reason, here we used real data from an actual catchment at Montseny but refer to it as “reference catchment” (RC0 hereafter), and avoided providing geographical details of its location and on whether it harbors *C. arnoldi* or not. Because of the high similarity in climate, lithology, vegetation, and topography of RC0 with other neighboring catchments (which might or might not harbor populations of *C. arnoldi*), we consider that any results and conclusions obtained by studying this catchment can be extrapolated to other sites at Montseny where *C. arnoldi* inhabits.

**Figure 2:**
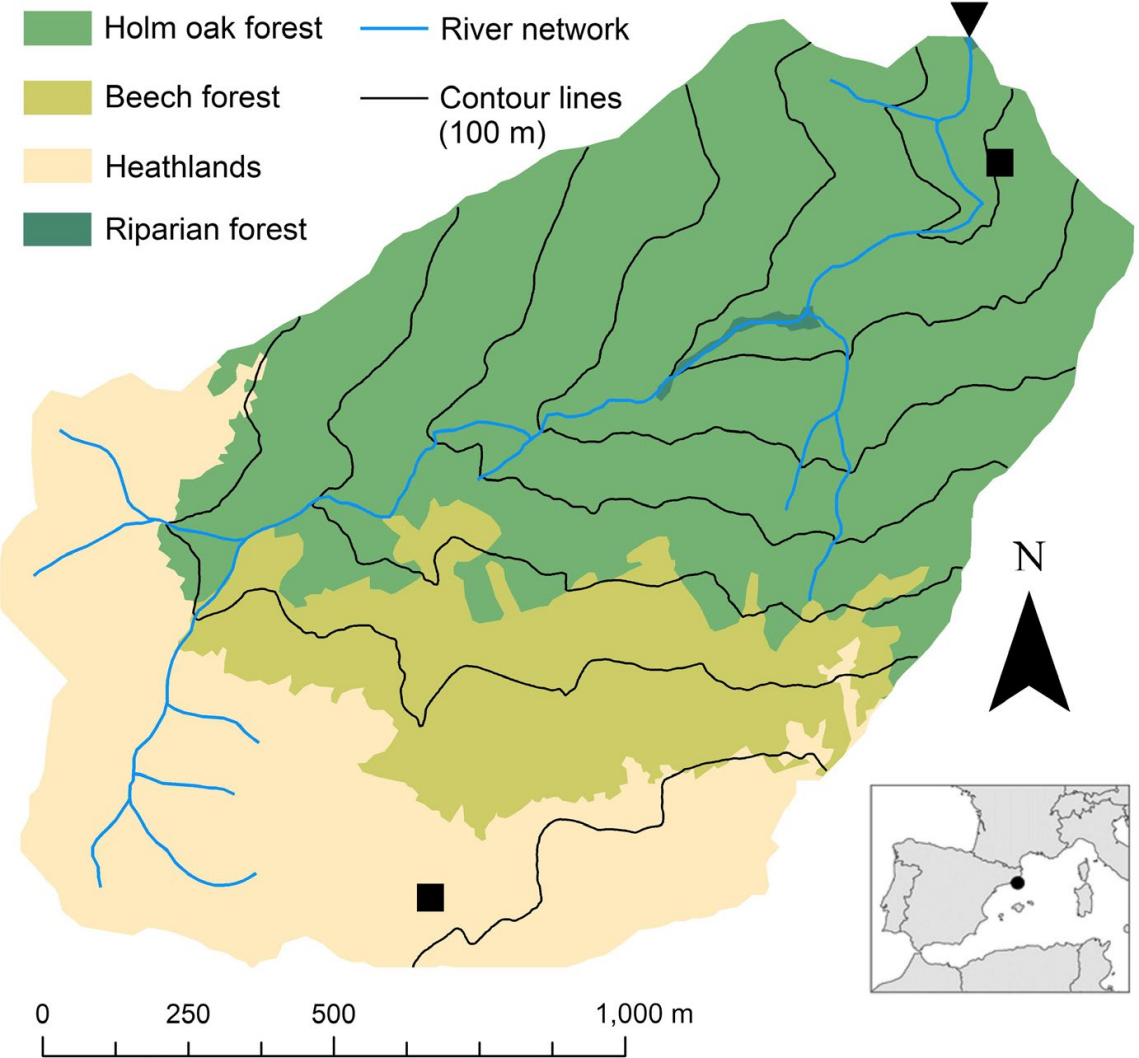
Reference study catchment site including vegetation covers, gridded climate data locations (black squares), and streamflow measurement location (inverted black triangle). The location of the catchment within Spain is shown in the inset

RC0 is a 2 km^2^ Mediterranean forest headwater catchment located in the Montseny Natural Park, NE Spain (Figure 2). The climate here is subhumid Mediterranean, with mild winters, wet springs and autumns, and dry summers. Mean annual air temperature is 11.5 ± 0.5°C (average ± *SD*) and total annual precipitation averages 983 ± 247 mm, less of 3% falling as snow (gridded data from the Meteorological Service of Catalonia for the period 1971–2005). Gentle slopes are present in the upper parts of the catchment, and steeper slopes are found in mid‐ and downstream valleys (Figure 2). Higher altitudes are dominated by heathlands primarily composed of heather (*Calluna vulgaris* and gramineae) and comprise 32.7% of the catchment. Holm oak (*Quercus ilex*) forest dominates the lower half (52.2%) of the catchment, whereas beech (*Fagus sylvatica*) forest occupies a 200 m stripe on the north‐facing slope between the heathlands and the holm oak forest and covers 15.1% of the catchment. Apart from low‐intensity grazing in the heathland‐dominated areas, there is currently no relevant human‐related activity in the catchment.

### 2.2 Streamflow and climate data

Streamflow was measured at the outlet of RC0 between January 2011 and December 2015 in a stilling pond using a 120° V‐notch weir equipped with a limnimetric scale. A pressure sensor system, consisting of a submerged Schlumberger Mini‐Diver sensor and a Schlumberger Baro‐Diver above the water surface, was used to record water levels every 15 min. Sensor water‐level data were tested against manual water‐level readings from the limnimetric scale, showing a strong correlation (*R* ^2^ = .98; *p* < .0001; *N* = 96). Subsequently, high‐frequency measurements were converted to streamflow through the specific stage‐flow rating curve for the location, and aggregated into daily data. A total of 1,618 daily streamflow observations were available, that is, 89% of the days within the 5‐year observed period. Streamflow will be denoted here by areally normalized units of mm/day (or mm/year where appropriate) for easy comparison to other sites. The proposed daily low and high streamflow event thresholds will be denoted by units of L/s, which are more intuitive for specific events in an ecological context.

Gridded climate data were computed at the Meteorological Service of Catalonia (SMC). Instrumental measurements of daily temperature (maximum and minimum) and precipitation were interpolated in space using a linear multiregression method (Altava‐Ortiz, Bladé, & Llasat Botija, 2010). The process considered altitude, latitude, longitude, and sea distance within different climatic clusters from more than 1,250 rain gauges and 660 temperature stations distributed over Catalonia for the 1970–2015 period. Climatic clusters were defined as a result of the maximization of monthly data spatial correlation in the entire region. Residual values (i.e., interpolated minus observed values) were geographically corrected using a kriging interpolation method, which took into account the spatial autocorrelation of the involved data. The interpolation was done for 1‐km^2^ grid cells. Two of the data points from the grid laid within the RC0 catchment area, one in the upper part of the catchment and the second one in the valley near the outlet (Figure 2). Daily averages of these two points (for both mean temperature and total precipitation) were calculated for the 5‐year period 2011–2015 and assumed to be representative of the whole catchment. In any case, there were only minor differences between the data series from these two grid points (data not explicitly shown).

### 2.3 Rainfall–runoff modeling: characterization and present‐day calibration

The rainfall–runoff model PERSiST (Futter et al., 2014) was used to simulate streamflow at the outlet of the RC0 catchment (inverted black triangle in Figure 2). PERSiST is a conceptual, semi‐distributed tool for simulating water fluxes, including streamflow, through physically based routing schemes at daily time steps. It involves a flexible framework based on a number of interconnected buckets within a mosaic of landscape units, which are specified by the modeler based on the conceptual perception of the runoff generation process. The model requires daily time series of air temperature and precipitation as input data. Parameter descriptions and other model details can be found elsewhere (Futter et al., 2014; Ledesma & Futter, 2017; Lupon et al., 2018).

The application of PERSiST to RC0 consisted of three landscape units, which corresponded to the catchment vegetation covers described above (holm oak forest, beech forest, and heathland) in appropriate proportions, and three soil buckets including a “quick” box that routed the water inputs from precipitation to the soil, a shallow “soil” layer for fast runoff responses, and a deeper “groundwater” layer for base flow regulation. The average of the gridded mean daily temperature and total daily precipitation from the two grid points located within the catchment generated at the SMC was used as input data. Gridded climate data products are a reliable alternative to instrumental measurements as inputs to rainfall–runoff models (Ledesma & Futter, 2017).

The model was calibrated for the 5‐year period with streamflow observations (2011–2015) using a combination of manual and automatic calibration techniques. Manual calibration has been proven as a robust method for obtaining acceptable simulations within the Integrated Catchment (INCA) family of models (Cremona, Vilbaste, Couture, Nõges, & Nõges, 2017; Futter et al., 2014; Ledesma, Köhler, & Futter, 2012), of which PERSiST is the common hydrological model. An initial manual calibration employing sensible values and thresholds based on expert judgement was used to approximate the dynamics and magnitude of the simulated streamflow to those of the observed streamflow. This step also helped identifying credible parameter ranges for a subsequent automated calibration procedure, which consisted on a Monte Carlo scheme that optimized the values of four calibration criteria (i.e., performance metrics): (i) the Nash–Sutcliffe (NS) efficiency index (important to fit high flows; Nash & Sutcliffe, 1970), (ii) the log(NS) (important to fit low flows), (iii) the relative volume differences (RVD) of simulated versus observed streamflow (important to ensure that cumulative modeled and observed streamflows are as similar as possible), and (iv) the ratio of observed to simulated variance (VAR) in streamflow values (important because automated calibration strategies tend to produce modeled series that are less variable than observed data) (Oni et al., 2016). The Monte Carlo process was carried out in 100 iterations of 1,000 runs each, and a single best‐performing parameter set was selected for final manual tuning.

Model parameter sensitivity was assessed using the 100 best‐performing parameter sets obtained in the automated Monte Carlo calibration. For each parameter, the ensemble of values from the 100 parameter sets was compared to a rectangular distribution using a Bonferroni‐corrected Kolmogorov–Smirnov (KS) test. A significant KS statistic (adjusted *p* < .01) implied that the posterior distribution was not rectangular and thus that streamflow simulations were sensitive to the specific parameter (Futter et al., 2014).

### 2.4 Selection of critical low and high streamflow thresholds for *Calotriton arnoldi*

*Calotriton arnoldi* has been very recently discovered, and at present, no direct observations exist that can provide critical threshold values for deleterious low or high streamflows for the species. Furthermore, an exhaustive bibliographic exploration did not return relevant results concerning the definition of threshold values for any amphibian species, which may have helped to constrain and compare our proposed low and high flow thresholds. Thus, here we propose threshold values based on knowledge of *C. arnoldi* biological cycle (larval and adult phases), feeding habits and survival strategy, and observation of the RC0 hydrological behavior. A daily low flow threshold of *Q*_low_ = 0.33 L/s was selected. At this streamflow value, downstream reaches near the RC0 catchment outlet show no superficial water flow because they have low slopes and are covered by sand, gravels, and pebbles (personal observation). Therefore, *Q*_low_ has been considered as an adequate working threshold for dry conditions.

No observation or model results exist for *C. arnoldi* to determine the risk of catastrophic drift for high flow thresholds. Studies investigating the related *C. asper* species have shown that intense precipitation events (>50 mm/day) cause catastrophic drift of larvae and adults, leading to reduced annual population numbers (Montori et al., 2012). In the RC0 and other catchments at Montseny, high flow peaks strongly depend on the antecedent conditions of humidity in the catchment soils (Àvila, Piñol, Rodà, & Neal, 1992). Precipitation events of 50 mm/day will result in very different streamflow responses depending on whether they occur in the dry (April to September) or wet season (October to March). Flow increase rate is thus the relevant variable to assess risk in the present case. To depict this, we generated the variable “flow difference in consecutive days” (*Q*_diff_), which was defined as the difference in observed streamflow between two consecutive days. Threshold values were defined as the 99th percentile of the *Q*_diff_ values for the whole year (*Q*_diff‐all_ = 97 L/s; *N* = 1,612) and for the dry season (*Q*_diff‐dry_ = 42 L/s; *N* = 776). All days above *Q*_diff‐all_ (or *Q*_diff‐dry_) were considered as single‐day events independently on whether they were followed by another day above *Q*_diff‐all_ (or *Q*_diff‐dry_) or not.

Besides optimizing the performance metrics described above (“hard calibration” criteria), the PERSiST calibration also aimed at simulating a number of days below *Q*_low_ as similar as possible to the observed number of days below *Q*_low_ (“soft calibration” criteria) (Lupon et al., 2018). Analogously, the calibration aimed at simulating a number of days above *Q*_diff‐all_ and *Q*_diff‐dry_ as similar as possible to the observed number of days above *Q*_diff‐all_ and *Q*_diff‐dry_.

### 2.5 Future climate and hydrological projections

Two emission scenarios from the Representative Concentration Pathways (RCP) were considered: the RCP4.5 (moderate carbon dioxide [CO_2_] emission scenario) and the RCP8.5 (extreme‐high CO_2_ emission scenario), to obtain near‐future (2031–2050) and far‐future (2081–2100) daily time series of temperature and precipitation at the same grid points used for generating inputs for streamflow calibration. Historical CO_2_ concentration values were used to obtain the reference period 1981–2000. Reference and future projected data were based on a statistical downscaling technique performed at the SMC (Altava‐Ortiz et al., 2010) and driven by a global set of daily simulations from the Max‐Planck Institute Earth System Model (MPI‐ESM; Giorgetta et al., 2013), which were available from the World Data Centre for Climate Portal CERA (https://cera-www.dkrz.de). Daily precipitation and temperature data from the two grid points used for calibration were again averaged prior using them as inputs in PERSiST to simulate future streamflows.

Four landscape scenarios were also considered to be combined with the climate scenarios in order to account for potential changes in vegetation cover proportions linked to the changes in climate. Based on the results presented by Peñuelas and Boada (2003), the following scenarios were considered: (i) Vegetation proportions are maintained as present; (ii) holm oak forest takes over 50% of the beech forest area and, in its turn, beech forest takes over 50% of the heathland area; (iii) holm oak forest takes over 50% of the beech forest area and the heathland area does not change; and (iv) holm oak forest takes over the whole catchment area (Table 1). The single best‐performing parameter set obtained during model calibration was used to simulate future streamflows for the 16 future scenarios (2 periods × 2 climates × 4 vegetation covers) and for the reference scenario.

**Table 1:** Summary of vegetation cover proportion scenarios

Scenario	Holm oak forest (%)	Beech forest (%)	Heathland (%)
(i) Vegetation as present	52.2	15.1	32.7
(ii) Beech increased	59.8	23.9	16.4
(iii) Heathland protected	59.8	7.6	32.7
(iv) Holm oak whole	100	0	0

### 2.6 Statistical analyses

Analysis of variance (ANOVA) was applied to estimate the relative contribution of period (near future and far future), climate scenario (RCP4.5 and RCP8.5), and vegetation cover scenario (Table 1) to the total variation in the number of days with daily simulated streamflow not reaching/exceeding the specified thresholds: *Q*_low_ = 0.33 L/s for low flows, and *Q*_diff‐all_ = 97 L/s (whole year) and *Q*_diff‐dry_ = 42 L/s (dry season) for flow increase events in consecutive days. This was done to disentangle the relative importance of climate versus vegetation cover in future hydrological extremes that might affect *C. arnoldi*. The fraction of total variation ascribed to each ANOVA component (i.e., period, climate scenario, or vegetation cover scenario) was equal to the sum of squares for that component divided by the total sum of squares from the ANOVA, following Futter et al. (2011) and Ledesma et al. (2013).

In order to investigate how the results would have changed if a different value of *Q*_low_ would have been selected, we obtained the number of simulated days below *Q*_low_ for each scenario (reference + 16 future scenarios) using five other values of *Q*_low_ (namely 2, 1, 0.5, 0.25, and 0.2 L/s). We considered that the conclusions of the study would hold if the simulated days below *Q*_low_ estimated for the reference and the future scenarios using different *Q*_low_ thresholds were linearly correlated with the analogous values using the selected *Q*_low_ of 0.33 L/s.

## 3 RESULTS

### 3.1 Streamflow model calibration and sensitivity analysis

**Figure 3:**
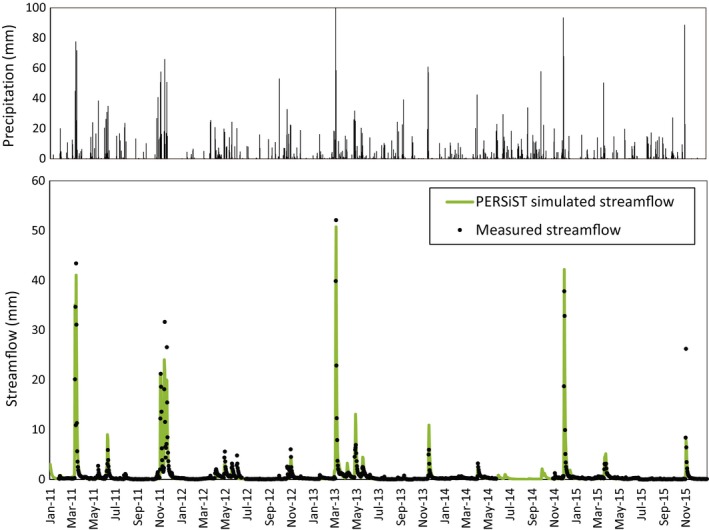
Daily observed and simulated streamflow (lower panel) and corresponding precipitation (upper panel) time series in the reference catchment

For the observation period (2011–2015), streamflow was dominated by low base flow. RC0 showed fast responses to some (but not all) precipitation events, which in the large storm cases generated as much as 52 mm/day of streamflow. A cross‐correlation analysis showed that the time lag that maximized the correlation between daily precipitation and daily observed streamflow was 1 day (Figure S1). PERSiST captured remarkably well this pattern including both the dynamics and the magnitude of streamflow and even the extremely high flow events, except for an intermediate event at the end of 2015 (Figure 3). The ratio of observed to simulated variance was 0.97, indicating just a slightly higher variance in the simulated flows. Cumulative observed streamflow was only slightly underestimated by the cumulative simulated streamflow (3%). The NS efficiency index was 0.89, indicating a very good fit of high flows. Finally, the log(NS) was 0.89, indicating a very good fit of low flows.

PERSiST was also able to simulate a very similar amount of days below and above the proposed daily low and high streamflow threshold values compared with the observed amount of days. The number of days in which streamflow was below *Q*_low_ in the observation record (*N* = 1,618) was 44. For the same record, the number of daily simulated streamflows below this threshold was 42. There were 16 observed single‐day events above *Q*_diff‐all_, whereas PERSiST simulated 14 single‐day events. For the *Q*_diff‐dry_ threshold, there were eight observed versus six simulated single‐day events.

Simulated streamflow was sensitive to four out of the 28 model parameters tested using the Bonferroni‐corrected KS test (Table S1). Three of the sensitive parameters related to the residence time of water in the “soil” and “groundwater” layers of the holm oak forest and heathland vegetation covers, with lower values producing better model performances. The other sensitive parameter was the so‐called “evapotranspiration adjustment” in the holm oak forest, which is used to limit evapotranspiration under dry conditions. Higher values of the evapotranspiration adjustment generated better model performances.

### 3.2 Projected future climate

**Figure 4:**
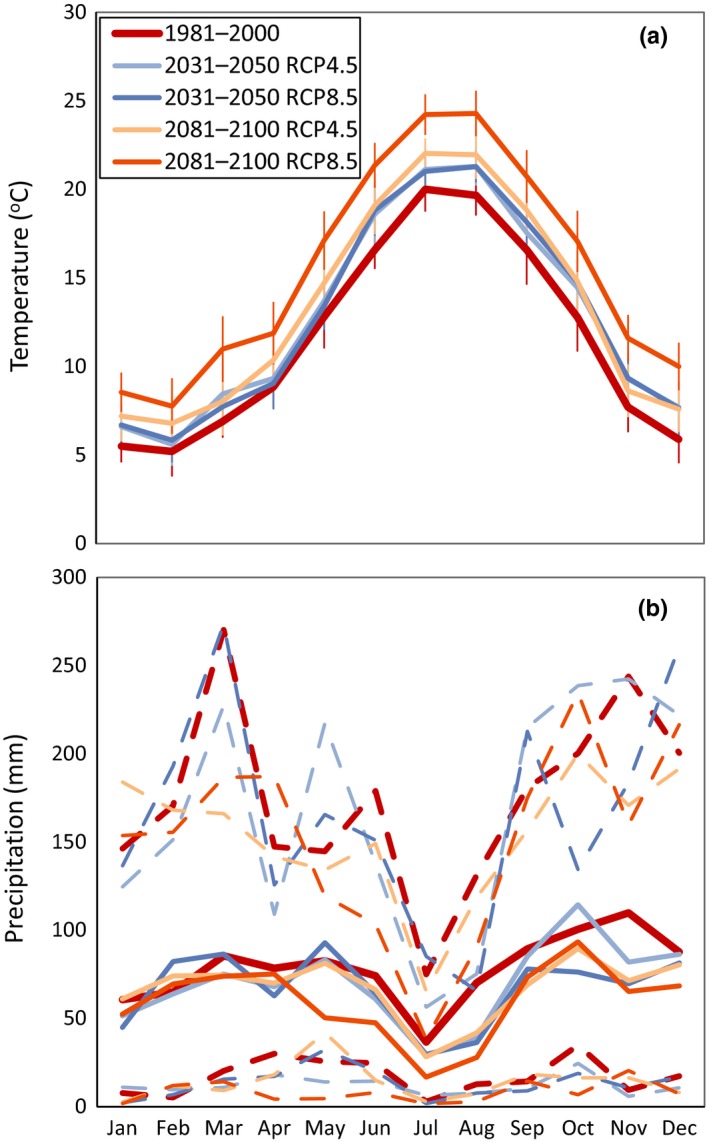
Monthly average temperature (a) and precipitation (b) for the reference period (1981–2000) and for Representative Concentration Pathway (RCP) scenarios 4.5 and 8.5 for near. (2031–2050) and far future (2081–2100). Error bars in (a) are standard deviations. Dotted light lines in (b) are 90th (above) and 10th (below) percentiles

According to both RCP4.5 and RCP8.5 scenarios, mean annual temperature in the near future (2031–2050) is projected to increase 1.3°C with respect to the mean annual temperature (11.6°C) of the reference period (1981–2000). In the far future (2081–2100), temperature will increase 1.8°C according to the RCP4.5 scenario and as much as 3.9°C according to the RCP8.5 scenario. In the near future, the increase will be more moderate in winter and spring months, whereas it will be more pronounced in summer and, especially, autumn (Figure 4a). The increase in temperature in the far future will be more evenly distributed throughout the year, although slightly less pronounced in winter and slightly more pronounced in summer.

Average annual precipitation will decrease with respect to the reference period value (941 mm/year) in all future scenarios. In the near future, RCP4.5 projected an 11% decrease, whereas RCP8.5 projected a 16% decrease (down to 805 mm/year). Similarly, RCP4.5 projected a 17% decrease in the far future, whereas RCP8.5 projected a 28% decrease for this period (i.e., down to 714 mm/year). In all cases, the decrease will be relatively more pronounced in the already dry summer months, whereas winter precipitation will be relatively similar to reference period precipitation (Figure 4b).

### 3.3 Projected future streamflow

**Figure 5:**
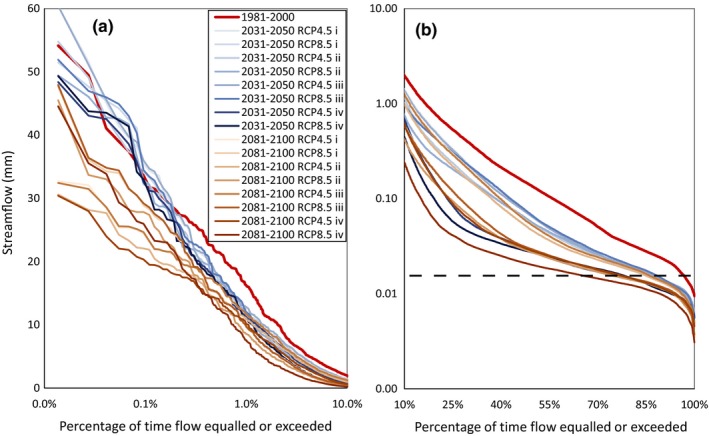
Flow duration curves for 0%–10% (a) and 10%–100% (b) time exceedance for each of the 16 scenarios considered (2 future periods × 2 climate scenarios × 4 vegetation cover scenarios) and for the reference period. Note the logarithmic scale in the *x*‐axis in (a) and the *y*‐axis in (b). Dotted line corresponds to the daily low flow threshold *Q*_low_ = 0.33 L/s

Daily extreme high flow events (*Q* > 30 mm/day) at RC0 will be as frequent in the near future as in the reference period (ca. 0.5% of the time) according to all scenarios (Figure 5a). On the contrary, all far‐future scenarios projected less frequency of these events. Daily streamflow events between 10 and 30 mm/day will be less frequent in both the near‐ and, especially, the far future. Intermediate and base flow conditions will yield lower stream water discharge than reference conditions according to all scenarios, that is, for any given exceedance time above 10%, streamflow magnitude will be lower in all cases (Figure 5b).

Consequently, all scenarios projected a significant overall decrease in average annual streamflow with respect to the reference period (346 mm/year), from a 21% decrease (near‐future RCP4.5 with vegetation as present) to as much as a 67% decrease (far‐future RCP8.5 with whole holm oak forest cover; Table 2). In parallel, the number of days with simulated streamflow lower than *Q*_low_ will dramatically increase with respect to the reference period (Table 2; Figure 5b). Most scenarios simulated streamflow below *Q*_low_ during 10%–20% of the time, with a maximum of 30% for the far‐future RCP8.5 scenario with whole holm oak forest cover, whereas for the reference period the same estimate was 2.6%. Far‐future and RCP8.5 scenarios will tend to be drier than their corresponding near‐future and RCP4.5 scenarios, respectively. Whole holm oak forest cover scenarios projected remarkably dry conditions, whereas scenarios with an increased beech forest cover or a protected heathland cover projected similar conditions, which were slightly drier than the corresponding vegetation as present scenarios.

**Table 2:** Number of days with daily simulated streamflow not reaching/exceeding the specified thresholds: *Q*_low_ = 0.33 L/s for low flows, and *Q*_diff‐all_ = 97 L/s (whole year) and *Q*_diff‐dry_ = 42 L/s (dry season) for flow increase events in consecutive days

Period	Climate scenario	Vegetation scenario	*Q*_low_ (days)	*Q*_low_ (events)	*Q*_diff‐all_ (days)	*Q*_diff‐dry_ (days)	*Q* (mm/year)
1981–2000	Reference	i	187	11	74	33	346
2031–2050	RCP4.5	i	758	31	63	25	272
2031–2050	RCP8.5	i	722	24	49	22	238
2031–2050	RCP4.5	ii	853	32	52	20	240
2031–2050	RCP8.5	ii	802	23	46	17	208
2031–2050	RCP4.5	iii	760	33	61	25	270
2031–2050	RCP8.5	iii	721	24	50	20	236
2031–2050	RCP4.5	iv	1,527	58	49	17	196
2031–2050	RCP8.5	iv	1,376	39	40	15	166
2081–2100	RCP4.5	i	854	31	54	26	238
2081–2100	RCP8.5	i	1,369	62	45	18	165
2081–2100	RCP4.5	ii	936	32	48	22	208
2081–2100	RCP8.5	ii	1,533	63	36	15	143
2081–2100	RCP4.5	iii	852	30	54	26	235
2081–2100	RCP8.5	iii	1,369	62	43	17	164
2081–2100	RCP4.5	iv	1,393	48	46	27	166
2081–2100	RCP8.5	iv	2,152	68	34	14	114

Note

Data are for each of the 16 scenarios considered (2 future periods × 2 climate scenarios × 4 vegetation cover scenarios) and for the reference period. Number of events with consecutive days below *Q*_low_ and annual average streamflow are also shown. All simulated days above *Q*_diff‐all_ or *Q*_diff‐dry_ were considered independent (i.e., single‐day events), and thus, the number of days reported corresponds to the number of events.

**Figure 6:**
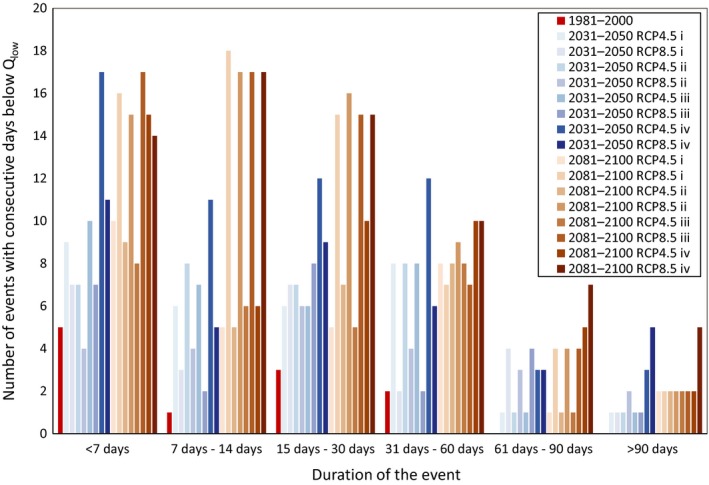
Duration and number of events with consecutive days below the daily low flow threshold *Q*_low_ = 0.33 L/s for each of the 16 scenarios considered (2 future periods × 2 climate scenarios × 4 vegetation cover scenarios) and for the reference period

In the reference period, most events with consecutive days below *Q*_low_ were shorter than a week and only two events were longer than a month (Figure 6). However, the number and length of these events will dramatically increase in the future, with all scenarios predicting at least one event with consecutive days below *Q*_low_ longer than 3 months (Table 2; Figure 6). The number and length of these events was especially magnified during the whole holm oak forest cover scenarios. In the near‐future scenarios, days below *Q*_low_ will be approximately homogeneously distributed over winter, summer, and autumn months, with lower proportions during spring (Table S2). In the far future, as in the reference period, days below *Q*_low_ will be more predominant during summer.

**Figure 7:**
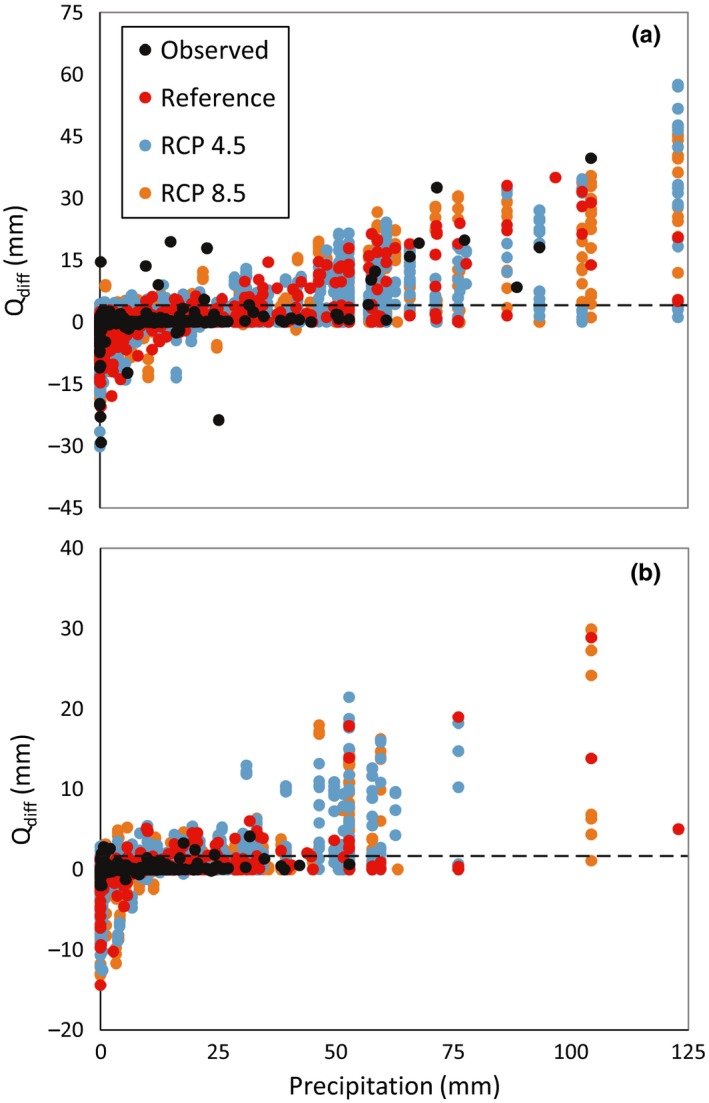
Scatter plots of “flow difference in consecutive days” (*Q*_diff_), versus daily precipitation for (a) the whole year and (b) the dry season (April to September) for future Representative Concentration Pathway (RCP) scenarios 4.5 and 8.5 and for the reference period. Dashed lines correspond to the threshold values defined as the 99th percentile of the *Q*_diff_ values for (a) the whole year (*Q*_diff‐all_ = 97 L/s), and for (b) the dry season (*Q*_diff‐dry_ = 42 L/s) using the observed data, which are also shown

During the reference period, maximum *Q*_diff_ was ca. 35 mm and the single‐day events larger than *Q*_diff‐all_ occurred mainly through November to March, typically the wettest and coldest months in Montseny. In all future scenarios, especially the RCP8.5 scenarios, the number of single‐day events above *Q*_diff‐all_ will decrease (Table 2) and concentrate in March and December. A number of single‐day events above *Q*_diff‐dry_ were slightly less than half of those above *Q*_diff‐all_ in all cases. Furthermore, *Q*_diff_ had no clear relationship with the amount of precipitation during the single‐day event for any of the scenarios or the observations, that is, the same precipitation amount (including high rainfall events) generated a wide range of *Q*_diff_ values, especially during the dry season (Figure 7).

The ANOVA (Table S3) revealed that vegetation cover scenarios explained more of the variance in the number of days with simulated streamflow below *Q*_low_ (50%) than the climate scenarios (11%) and the period considered (21%) (Table 3). In contrast, climate scenarios explained most of the variance in the number of simulated single‐day events above *Q*_diff‐all_ (47%) and *Q*_diff‐dry_ (53%), whereas vegetation cover scenarios explained a moderate amount of variance. The period considered explained 17% of the variance in the number of simulated single‐day events above *Q*_diff‐all_ and a negligible variance of above simulated *Q*_diff‐dry_ single‐day events (Table 3). Simulated days below *Q*_low_ using a range of *Q*_low_ thresholds were linearly correlated with the simulated days below *Q*_low_ from the selected *Q*_low_ of 0.33 L/s, as obtained from the reference and the 16 future scenarios (Figure S2).

**Table 3:** Relative contribution of period (near‐ and far‐future), climate scenario (RCP4.5 and RCP8.5), and vegetation cover scenario (Table 1) to the total variation in the number of days with daily simulated streamflow not reaching/exceeding the specified thresholds: *Q*_low_ = 0.33 L/s for low flows, and *Q*_diff‐all_ = 97 L/s (whole year) and *Q*_diff‐dry_ = 42 L/s (dry season) for flow increase events in consecutive days

Component	*Q*_low_ (%)	*Q*_diff‐all_ (%)	*Q*_diff‐dry_ (%)
Period	21	17	0.3
Climate scenario	11	47	53
Vegetation cover scenario	50	33	22
Model	82	97	76
Residual	18	3	24

## 4 DISCUSSION

### 4.1 Overall streamflow model performance and catchment hydrological behavior

PERSiST reproduced remarkably well the flow magnitude and dynamics at RC0, as well as the number of days below *Q*_low_ and number of single‐day events above *Q*_diff‐all_ and *Q*_diff‐dry_. Combining specific streamflow characteristics (here the specific thresholds *Q*_low_, *Q*_diff‐all_, and *Q*_diff‐dry_) with commonly used model performance metrics (here NS, log(NS), RVD, and VAR) during calibration procedures is recommended to achieve accurate streamflow simulations for practical applications (Pool, Vis, Knight, & Seibert, 2017; Vis, Knight, Pool, Wolfe, & Seibert, 2015). An accurate model simulation was indeed achieved in the present application, suggesting that a robust parameterization was used for future simulations of streamflow.

Observed and simulated patterns of streamflow at RC0 indicate that the catchment is very flashy, that is runoff generation is notably fast, and the sensitivity analysis suggested that streamflow is mostly sensitive to residence time of soil water and groundwater. This suggests that short water residence times related to topography and seasonality drive runoff generation. Seasonality refers to the different hydrological behavior during wet versus dry conditions as, for example, high flow peaks strongly depend on the antecedent soil wetness (Àvila et al., 1992). Here, streamflow was also sensitive to the evapotranspiration adjustment parameter used to limit evapotranspiration under dry conditions, which indicates that during these periods there is low hydrological connectivity between soils and the stream and precipitation inputs contribute to increase catchment water storage rather than to streamflow generation or evapotranspiration. Consistently, precipitation was only weakly related to *Q*_diff_ during the observation period and this behavior was well captured by the future simulations, especially during the dry season (Figure 7).

### 4.2 Vulnerability of *Calotriton arnoldi* to future low streamflows

Establishing critical streamflow thresholds for amphibian species is difficult, especially for endangered species because observations and data are scare, but we consider our proposed *Q*_low_ value an adequate working threshold. Nevertheless, small changes in the *Q*_low_ proposed here would have yielded the same results in relative terms (Figure S2), and therefore, the conclusions discussed below apply to a range of low streamflow values that could have been selected as thresholds.

Our simulations indicate that projected climate and vegetation cover changes will severely threaten *C. arnoldi* survival by increasing the frequency and duration of very low streamflows, which may lead to periods of prolonged lack of surficial flow. Previous studies with *C. asper* reported that climate change severely reduced the species distribution range because of the reduced possibility of dispersal, leading to a loss of genetic diversity (de Pous, Montori, Amat, & Sanuy, 2016). Our results suggest that *C. arnoldi* will need to migrate in search of suitable habitats during periods of drought and the lack of suitable sites may lead to important losses both in population individuals and genetic diversity. Furthermore, in case of dwindling populations of *C. arnoldi*, low genetic diversity existing in the remaining populations constitutes an additional bottleneck that may lead to extinction (Valbuena‐Ureña et al., 2013).

Critical low streamflow projections were not very different considering the moderate (RCP4.5) or the extreme (RCP8.5) scenarios, whereas vegetation cover had an important role in determining the low streamflow hydrology of the catchment. Scenarios in which the open heathlands are maintained irrespective of the tree species covering the rest of the catchment present considerably lower number of days below *Q*_low_ than scenarios where holm oak forest covered the whole catchment. Holm oak forests have water demands that are significantly larger than those of open heathlands and can transpire even during dry periods (Ladekarl, Rasmussen, Christensen, Jensen, & Hansen, 2005; Paço et al., 2009), and so evapotranspiration will make up a significantly larger proportion of the water balance under whole holm oak forest cover scenarios. Our simulations thus suggest that the potential change in vegetation cover toward an expansion of holm oak forests at the expense of beech forests and heathlands will be more important than climate change itself in determining critical low streamflow conditions in the Montseny mountains.

### 4.3 Vulnerability of *Calotriton arnoldi* to future high streamflows

Risk of catastrophic drift in *C. asper* was related to precipitation events larger than 50 mm/day (Colomer et al., 2014; Montori et al., 2012). In these studies, no indication of associated streamflows was provided as the investigated catchments were ungauged. At RC0, the threshold based on actual streamflow increase rate better describes the potential impact of high flows on *C. arnoldi* or other species, as the relationship between precipitation and streamflow is quite loose (Figure 7). During the dry season (April to September), *Q*_diff_ values were noticeably lower than those in the wet season and single‐day events above *Q*_diff‐dry_ concentrated in April and May, both in the reference and future scenarios (data not explicitly shown). The occurrence of these flashy events during spring may have deleterious consequences because this is a sensitive period in the *C. arnoldi* life cycle (reproduction occurs at this time), and drift risk is larger for eggs and larvae than for adults. Immature individuals of the related *C. asper* species can inhabit the terrestrial environment, and this represents a refuge that might help recovering population sizes after extreme streamflow increase events (Colomer et al., 2014; Montori et al., 2012). By contrast, immature individuals of *C. arnoldi* do not inhabit the terrestrial surroundings and remain in the aquatic environment until sexual maturity, which makes this species more vulnerable to floods and high water velocities. Nevertheless, our simulations show that the number of single‐day events above *Q*_diff‐dry_ will decrease in the future and so the potential risk of high streamflow events for *C. arnoldi* during its sensitive life cycle period will be reduced, as well as the potential risk of catastrophic drift overall.

In contrast to the importance of vegetation cover scenarios to explain the number of days below *Q*_low_, climate scenarios appear to explain most of the variance in the number of single‐day events above *Q*_diff‐all_ and, especially, above *Q*_diff‐dry_. RCP8.5 scenarios are extreme‐high CO_2_ emission scenarios in which temperatures are predicted to be higher and large rainfall events more sporadic than the corresponding RCP4.5 scenarios. These conditions will limit the opportunities for generation of high streamflow increase events due to the combination of increased evapotranspiration and lower probability of large rainfall events occurring under pre‐existent wet conditions.

### 4.4 Implications for conservation and management

As discussed by O'Driscoll et al. (2018), climate impact assessment studies, such as the one presented here, can be interpreted in qualitative terms based on alternative scenarios of internally consistent narratives, or “storylines,” which are based on a restricted set of possible sources of uncertainty and thus are representations of a subset of all possible futures (Cremona et al., 2017; van Vuuren, Vries, Beusen, & Heuberger, 2008). In our case, combining an educated selection of critical streamflow thresholds based on hydrological observations and *C. arnoldi* ecological requirements, with moderate (e.g., RCP4.5) and extreme (RCP8.5) climate scenarios, and four plausible vegetation cover scenarios ensured that a wide range of possible futures was covered. These relevant storylines on possible futures offer a starting point for decision‐making regarding *C. arnoldi* management in the context of the LIFE‐Tritó Montseny project that can guide future actions and research.

Our study shows that predicted future low streamflow conditions will pose a severe threat for the survival of existing *C. arnoldi* populations, or the viability of those planned to be reintroduced. This thread will be mostly driven by the hydrological consequences of the expected expansion of holm oak forests. In contrast, the risk of catastrophic drift due to high streamflow events will be reduced, especially under high RCP emission scenarios. Therefore, we recommend consideration of future potential changes in landscape (e.g., changes in vegetation cover) and not only predicted changes in climate variables in simulations of future streamflows using rainfall‐runoff models.

This work may help to take management actions (e.g., limit the expansion of the holm oak forest) for ameliorating the *C. arnoldi* newt habitat. This is relevant because very few case studies have up to now examined the effects of hydrological regimes on amphibian populations (Cayuela, Besnard, Béchet, Devictor, & Olivier, 2012) or the efficacy of landscape management practices devised to help the conservation of such populations (Hartel, Băncilă, & Cogălniceanu, 2011; Shoo et al., 2011). We used a holistic approach that brings together different scientific disciplines (hydrological modeling, climatology, biology, and ecology) for providing a basis for *C. arnoldi* management and conservation strategies. This type of approach is encouraged for formulating science‐based, sustainable management of both amphibian species and ecosystems.

## CONFLICT OF INTEREST

None declared.

## AUTHORS' CONTRIBUTIONS

JLJL and AA conceived the idea, designed the methodology, proposed the structure, and wrote the paper. JLJL performed the hydrological modeling and analyzed the data. AM provided support on the *Calotriton arnoldi* ecology and contributed to the writing. VA, AB, and JC carried out the climate modeling. All authors contributed critically to the drafts and gave final approval for publication.

## Data Availability

All data associated with the paper can be accessed through figshare at https://figshare.com/articles/Ledesma_et_al_2019_ECE_Dataset_xlsx/8869598 (https://doi.org/10.6084/m9.figshare.8869598).
