# Supplementary material for: Future hydrological constraints of the Montseny brook newt (Calotriton arnoldi) under changing climate and vegetation cover
Source: Ecol Evol. 2019 Aug 1;9(17):9736–47. doi: 10.1002/ece3.5506 (PMC6745664; doi:10.1002/ece3.5506)
Supplement: Supplementary file 1 [file ECE3-9-9736-s001.pdf]

Supporting Information

Future hydrological constraints of the Montseny brook newt (*Calotriton arnoldi*) under changing climate and vegetation cover

José L. J. Ledesma[1,2]*, Albert Montori[3], Vicent Altava-Ortiz[4], Antonio Barrera-Escoda[5], Jordi Cunillera[5], Anna Àvila[6]

[1]Centre for Advanced Studies of Blanes, Spanish National Research Council, Blanes, Spain

[2]Department of Aquatic Sciences and Assessment, Swedish University of Agricultural Sciences, Uppsala, Sweden

[3]GRENP (Grup de Recerca de l'Escola de la Natura de Parets del Vallès), Life-tritó del Montseny, Diputació de Barcelona, Parets del Vallès, Spain

[4]Department of Applied Research and Modelling, Meteorological Service of Catalonia, Barcelona, Spain

[5]Department of Climatology, Meteorological Service of Catalonia, Barcelona, Spain

[6]CREAF, Campus Universitat Autonoma Barcelona, Bellaterra, Spain

Correspondence: José L. J. Ledesma. E-mail: jose.ledesma@ceab.csic.es

Table of Contents

Figure S1. Cross-correlation between precipitation and streamflow S2

Table S1. Hydrological model sensitivity analysis S3

Table S2. Seasonal distribution of number of days below Q_{low} S4

Table S3. ANOVA estimates S5

Figure S2. Correlation between simulated days below different Q_{low} thresholds S6

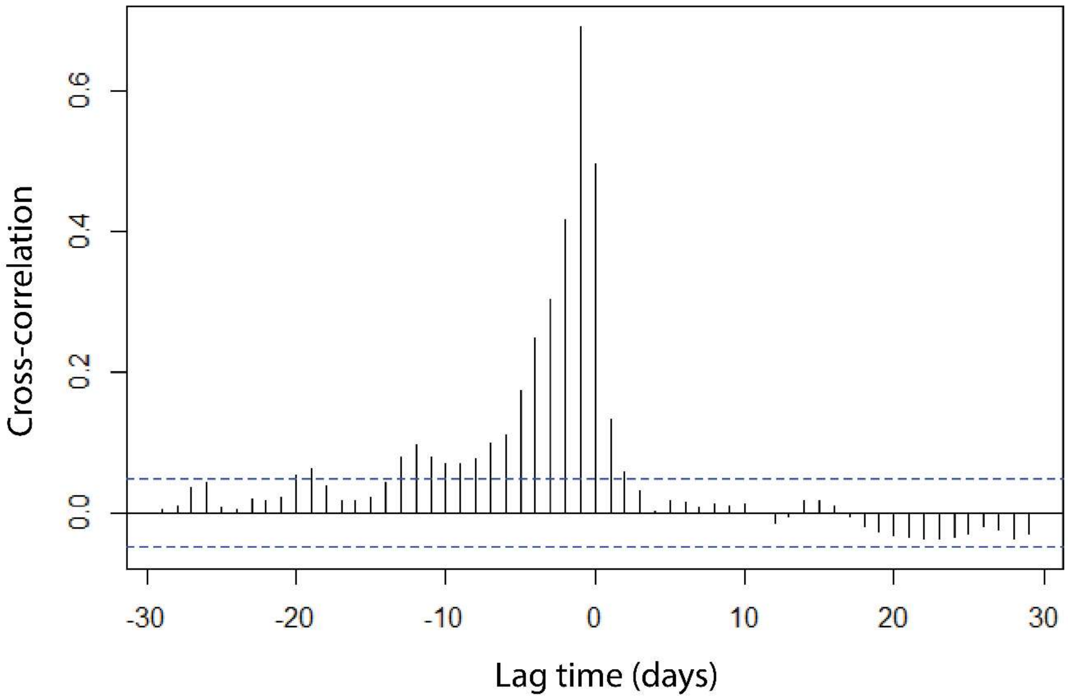

Figure S1. Cross-correlation between daily precipitation and daily observed streamflow. The correlation was strongest when lag time = -1 days, meaning that daily streamflow correlated most strongly with the preceding day precipitation.

Table S1. List of PERSiST parameters, including parameter ranges, tested for sensitivity in the application to the reference catchment at Montseny. Model parameter sensitivity was assessed using the 100 best performing parameter sets obtained in an automated Monte Carlo calibration, which was carried out in 100 iterations of 1000 runs each. For each parameter, the ensemble of values from the 100 parameter sets was compared to a rectangular distribution using a Bonferroni corrected Kolmogorov-Smirnov (KS) test, i.e. an adjusted p-value was obtained and it is shown in the table. A significant KS statistic (adjusted $p<0.01$) implied that the posterior distribution was not rectangular and thus that streamflow simulations were sensitive to the specific parameter. For a full description of model parameters, the reader is referred to Futter et al. (2014) and Lupon et al. (2018). 'Time constant' parameters represent residence time of water in each specified soil box as a proxy for the hydrological conductivity of that soil box.

**Parameter**	**Units**	**Min**	**Max**	**KS test**	**Sensitive**
a (flow velocity multiplier)	m^{-2}	0.15	0.45	13.89	
b (flow velocity exponent)	-	0.5	0.9	3.33	
Rain multiplier for *Evergreen forest*	-	0.9	1.1	0.02	
Rain multiplier for *Deciduous forest*	-	0.9	1.1	10.13	
Rain multiplier for *Heathland*	-	0.9	1.1	4.78	
Degree day evapotranspiration for *Evergreen forest*	mm °C^{-1} day^{-1}	0.15	0.25	9.55	
Degree day evapotranspiration for *Deciduous forest*	mm °C^{-1} day^{-1}	0.2	0.35	3.85	
Degree day evapotranspiration for *Heathland*	mm °C^{-1} day^{-1}	0.1	0.25	6.03	
Growing degree threshold for *Evergreen forest*	°C	0	3	0.69	
Growing degree threshold for *Deciduous forest*	°C	1	5	2.29	
Growing degree threshold for *Heathland*	°C	0	2	0.48	
Canopy interception for *Evergreen forest*	mm day^{-1}	0.25	1	1.44	
Canopy interception for *Deciduous forest*	mm day^{-1}	0.5	1.25	1.98	
Drought runoff fraction for *Evergreen forest, Soil layer*	-	0	0.2	0.01	
Drought runoff fraction for *Deciduous forest, Soil layer*	-	0	0.2	6.54	
Drought runoff fraction for *Heathland, Soil layer*	-	0	0.2	0.60	
Time constant for *Evergreen forest, Quick layer*	days	1.1	1.5	0.88	
Time constant for *Deciduous forest, Quick layer*	days	1.1	1.5	13.29	
Time constant for *Heathland, Quick layer*	days	1.1	1.5	11.88	
Time constant for *Evergreen forest, Soil layer*	days	1	3	<0.0001	YES
Time constant for *Deciduous forest, Soil layer*	days	1	3	3.09	
Time constant for *Heathland, Soil layer*	days	1	3	<0.0001	YES
Time constant for *Evergreen forest, Groundwater layer*	days	2	50	<0.0001	YES
Time constant for *Deciduous forest, Groundwater layer*	days	4	50	0.03	
Time constant for *Heathland, Groundwater layer*	days	4	50	1.33	
Evapotranspiration adjustment for *Evergreen Forest, Soil layer*	-	0	3	<0.0001	YES
Evapotranspiration adjustment for *Deciduous forest, Soil layer*	-	0	3	7.14	
Evapotranspiration adjustment for *Heathland, Soil layer*	-	0	3	0.02	

Table S2. Distribution over annual seasons of the number of days with daily simulated streamflow below the specified threshold for low flows (Q_{low} = 0.33 L s^{-1}) for the reference period (1981-2000) and for Representative Concentration Pathway (RCP) scenarios 4.5 and 8.5 for near- (2031-2050) and far-future (2081-2100).

Period	Climate scenario	Winter	Spring	Summer	Autumn
1981-2000	Reference	32%	10%	50%	9%
2031-2050	RCP 4.5	20%	10%	36%	33%
2031-2050	RCP 8.5	30%	11%	25%	35%
2081-2100	RCP 4.5	23%	9%	41%	28%
2081-2100	RCP 8.5	12%	13%	51%	24%

Table S3. Estimates from the analyses of variance (ANOVAs) that computed the relative contribution of period (near- and far-future), climate scenario (RCP4.5 and RCP8.5), and vegetation cover scenario (Table 1) to the total variation in the number of days with daily simulated streamflow not reaching/exceeding the specified thresholds: (a) Q_{low} = 0.33 L s^{-1} for low flows, and (b) $Q_{diff\text{-}all}$ = 97 L s^{-1} (whole year) and (c) $Q_{diff\text{-}dry}$ = 42 L s^{-1} (dry season) for flow increase events in consecutive days.

(a) Q_{low}

Component	Sum of Squares	F Ratio	Prob>F	Variation explained
Period	539858	11.4	0.007	20.8%
Climate scenario	278520	5.9	0.036	10.7%
Vegetation scenario	1301990	9.2	0.003	50.2%
Model	2120368	9.0	0.002	81.8%
Residual	471836	-	-	18.2%

(b) $Q_{diff\text{-}all}$

Component	Sum of Squares	F Ratio	Prob>F	Variation explained
Period	156	61.9	<0.0001	16.7%
Climate scenario	441	174.7	<0.0001	47.2%
Vegetation scenario	311	41.1	<0.0001	33.3%
Model	909	72.0	<0.0001	97.3%
Residual	25			2.7%

(c) $Q_{diff\text{-}dry}$

Component	Sum of Squares	F Ratio	Prob>F	Variation explained
Period	1	0.1	0.716	0.3%
Climate scenario	156	21.9	0.001	53.2%
Vegetation scenario	65	3.1	0.079	22.2%
Model	223	6.2	0.007	75.7%
Residual	71			24.3%

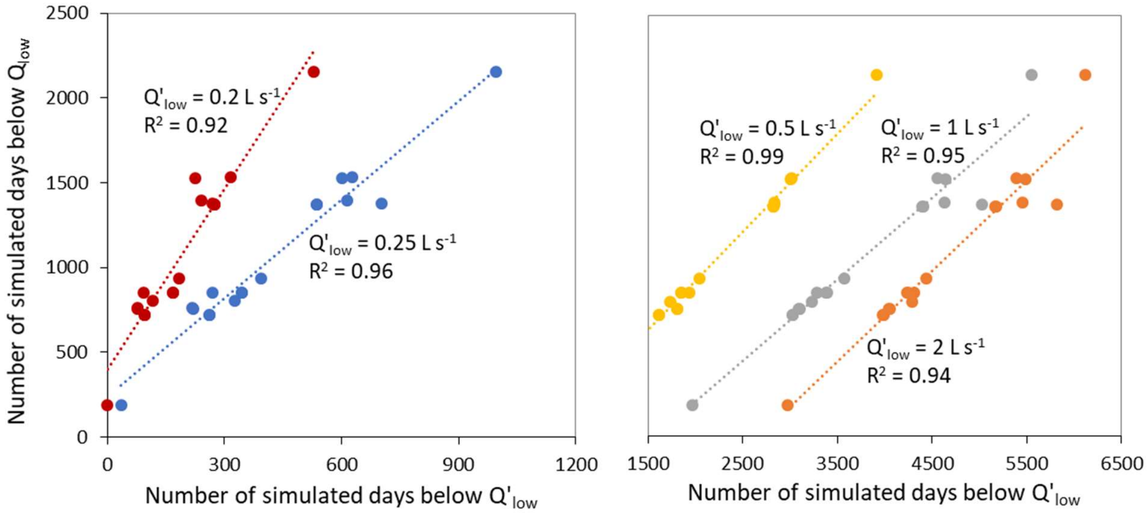

Figure S2. Number of simulated days below the selected low flow threshold Q_{low} = 0.33 L s^{-1} versus the number of simulated days below different values of Q_{low} (i.e. Q'_{low} = 2, 1, 0.5, 0.25, and 0.2 L s^{-1}) as obtained from the 17 scenarios considered in the study, i.e. the reference scenario and the 16 future scenarios (2 future periods x 2 climate scenarios x 4 vegetation cover scenarios). The five tested values of Q'_{low} are plotted in two panels for better visualization.
